# Exploiting the Acceleration Voltage Dependence of EMCD

**DOI:** 10.3390/ma14051314

**Published:** 2021-03-09

**Authors:** Stefan Löffler, Michael Stöger-Pollach, Andreas Steiger-Thirsfeld, Walid Hetaba, Peter Schattschneider

**Affiliations:** 1 University Service Centre for Transmission Electron Microscopy, TU Wien, Wiedner Hauptstraße 8-10/E057-02, 1040 Wien, Austria; michael.stoeger-pollach@tuwien.ac.at (M.S.-P.); andreas.steiger-thirsfeld@tuwien.ac.at (A.S.-T.); peter.schattschneider@tuwien.ac.at (P.S.); 2 Max Planck Institute for Chemical Energy Conversion, Stiftstraße 34-36, 45470 Mülheim an der Ruhr, Germany; hetaba@fhi-berlin.mpg.de; 3 Institute of Solid State Physics, TU Wien, Wiedner Hauptstraße 8-10/E138-03, 1040 Wien, Austria

**Keywords:** EMCD, TEM, EELS, magnetism, acceleration voltage

## Abstract

Energy-loss magnetic chiral dichroism (EMCD) is a versatile method for measuring magnetism down to the atomic scale in transmission electron microscopy (TEM). As the magnetic signal is encoded in the phase of the electron wave, any process distorting this characteristic phase is detrimental for EMCD. For example, elastic scattering gives rise to a complex thickness dependence of the signal. Since the details of elastic scattering depend on the electron’s energy, EMCD strongly depends on the acceleration voltage. Here, we quantitatively investigate this dependence in detail, using a combination of theory, numerical simulations, and experimental data. Our formulas enable scientists to optimize the acceleration voltage when performing EMCD experiments.

## 1. Introduction

Circular dichroism in X-ray Absorption Spectroscopy (XAS) probes the chirality of the scatterer, related either to a helical arrangement of atoms or to spin polarized transitions as studied in X-ray Magnetic Circular Dichroism (XMCD). Before the new millenium, it was considered impossible to see such chirality in electron energy-loss spectrometry (EELS). On the other hand, the formal equivalence between the polarization vector in XAS and the scattering vector in EELS tells us that any effect observable in XAS should have its counterpart in EELS. For instance, anisotropy in XAS corresponds to anisotropy of the double differential scattering cross section (DDSCS) in EELS. A well known example is the directional prevalence of either $s\to \pi^{*}$ and $s\to \sigma^{*}$ transitions in the carbon K-edge of graphite, depending on the direction of the scattering vector [1,2].

In XMCD, the polarization vector is helical—a superposition of two linear polarization vectors $e_{x}\pm ie_{y}$ orthogonal to each other—resembling a left- and right-handed helical photon, respectively. However, what is the counterpart of photon helicity in EELS?

In 2002, one of the authors and their postdoc speculated about what the counterpart of photon helicity could be in EELS—an arcane issue at the time. This led to a keen proposal to study spin polarized transitions in the electron microscope [3]. Closer inspection revealed that in EELS, a superposition of two scattering vectors orthogonal to each other with a relative phase shift of ±π/2 is needed, exactly as the formal similarity with XMCD dictated. This, in turn, called for a scattering geometry that exploits the coherence terms in the DDSCS [4,5]. These insights led to the CHIRALTEM project [6].

The multidisciplinary team elaborated the appropriate geometry for the analysis of ionization edges in the spirit of XMCD. The first EELS spectrum was published in 2006 [7]. In that paper, the new method was baptized EMCD—Electron (Energy Loss) Magnetic Chiral Dichroism—in analogy to XMCD. The term “chiral” was deliberately chosen instead of “circular” because the chirality of electronic transitions was to be detected, and because there is no circular polarization in EELS. The experiment confirmed that the physics behind EMCD is very similar to the physics of XMCD. Rapid progress followed: consolidation of the theory [8,9], optimization of experimental parameters [10], dedicated simulation software [11,12], and spatial resolution approaching the nm [13,14] and the atomic scale [15,16,17,18,19,20,21,22,23].

A genuine feature of EMCD is the ability to probe selected crystallographic sites [18,24], e.g., in Heusler alloys [25], ferrimagnetic spinels [26], or perovskites [27,28]. The high spatial resolution of the method allows the study of nanoparticles [14], 3d–4f coupling in superlattices [29], specimens with stochastically oriented crystallites and even of amorphous materials [30]. EMCD has also been used to investigate spin polarization of non-magnetic atoms in dilute magnetic semiconductors [31], magnetic order breakdown in MnAs [32], GMR of mixed phases [33] and magnetotactic bacteria [34]. A key experiment on magnetite, exploiting the combination of atomic resolution in STEM with the site specificity showed the antiferromagnetic coupling of adjacent Fe atoms directly in real space [16]. An overview of EMCD treating many aspects of anisotropy and chirality in EELS can be found in [35].

To date, EMCD measurements have predominantly been performed at the highest available acceleration voltages—typically 200 keV to 300 keV—which has several advantages such as better resolution, a larger inelastic mean free path, and optimal detector performance resulting in a reasonable signal-to-noise ratio. However, by limiting oneself to a specific acceleration voltage and hence electron energy, EMCD cannot be used to its full potential.

One example where choosing a lower acceleration voltage can be tremendously helpful is the reduction or avoidance of beam damage [36,37,38,39]. Another is the investigation of the magnetization dependence: in a TEM, the sample is placed inside the objective lens with a typical field strength of the order of 2 T for 200 keV electrons. By changing the acceleration voltage, the objective lens field applied at the sample position is changed as well [40], thereby enabling magnetization-dependent investigations. This can even be used to drive magnetic field induced phase transitions [27]. Moreover, EMCD is strongly affected by elastic scattering, and, hence, thickness and sample orientation [8,11,25,41]. Therefore, changing the electron energy and therefore the details of the elastic scattering processes enables EMCD measurements even at a thickness and orientation where no significant EMCD effect is observable at a high acceleration voltage. This proposition is corroborated by early numerical simulations [42], which to our knowledge have not been followed up on or widely adopted by the community.

## 2. Results

### 2.1. Theory

The general formula governing EMCD has already been outlined in the original publications theoretically predicting the effect and demonstrating it experimentally [3,7]. Detailed ab initio studies soon followed [8]. However, those formulations all aimed at very high accuracy; none of them gave a simple, closed form to quickly calculate the EMCD effect and easily see the influence parameters such as, e.g., the acceleration voltage have on the outcome. Recently, Schneider et al. [41] published such a formula; however, they neglected any elastic scattering the beam can undergo after an inelastic scattering event by approximating the outgoing wave by a simple plane wave.

Here, we present a derivation of a simple formula taking into account elastic scattering both before and after the inelastic scattering event. In the process, we will make four major assumptions:

1. We limit the derivation to an incident three-beam and outgoing two-beam case in the zero-order Laue zone of a sample that is single-crystalline in the probed region with a centro-symmetric crystal structure;
2. We assume that the sample is a slab of thickness *t* with an entrance and an exit plane essentially perpendicular to the beam propagation axis;
3. We assume that the inelastic scattering process is at least four-fold rotationally symmetric around the optical axis and that the characteristic momentum transfer $q_{e}$ is much smaller than the chosen reciprocal lattice distance |G|. This implies that the inelastic scattering in the chosen geometry is only dependent on the scattering atom’s spin-state, but not influenced significantly by any anisotropic crystal field;
4. We assume that the atoms of the investigated species are homogeneously distributed along the beam propagation axis and that G·x=2mπ,m∈Z for all atom positions x and the chosen lattice vector G.

Assumption 1 comes from the conventional EMCD setup: the (crystalline) sample is tilted into systematic row condition and the detector is placed on (or close to) the Thales circle between neighboring diffraction spots. In a symmetric systematic row condition, the strongest diffraction spots are the central one (0) and the two diffraction spots at −G,G, which have the same intensity. Any higher-order diffraction spots are comparatively weak and will therefore be neglected.

To understand the reason behind the outgoing two-beam case, we follow the reciprocity theorem [43,44]. A (point-like) detector in reciprocal space detects exact plane-wave components. If we trace those back to the exit plane of the sample, we can expand them into Bloch waves. For the typical EMCD detector positions, they correspond exactly to the Bloch waves we get in a two-beam case (where the Laue circle center is positioned somewhere along the bisector of the line from 0 to G.

The probability of measuring a particular state $|\psi_{\mathrm{out}}\rangle$ (a “click” in the detector corresponding to a plane wave at the exit plane of the sample) given a certain incident state $|\psi_{\mathrm{in}}\rangle$ (a plane wave incident on the entry plane of the sample) is given by Fermi’s Golden rule [45,46,47,48,49]:

(1) $$p=\sum_{I,F}p_{I}\left(1-p_{F}\right)\langle \psi_{\mathrm{out}}|\langle F|\hat{V}|I\rangle |\psi_{\mathrm{in}}\rangle \langle \psi_{\mathrm{in}}|\langle I|{\hat{V}}^{†}|F\rangle |\psi_{\mathrm{out}}\rangle \delta \left(E_{F}-E_{I}-E\right),$$

where I,F run over all initial and final states of the sample, $p_{I},p_{F}$ are their respective occupation probabilities, $E_{I},E_{F}$ are their respective energies, *E* is the EELS energy loss, and $\hat{V}$ is the transition operator. In momentum representation, $\hat{V}$ for a single atom is given by

(2) $$\langle \tilde{k}\left|\hat{V}\right|k\rangle =\frac{e^{iq\;\cdot \;\hat{R}}}{q^{2}}\;\mathrm{with}\;q=k-\tilde{k}.$$

With the mixed dynamic form factor (MDFF) [45,49,50,51],

(3) $$S\left(q,q^{\prime },E\right)=\sum_{I,F}p_{I}\left(1-p_{F}\right)\langle \tilde{k}|\langle F|e^{iq\;\cdot \;\hat{R}}|I\rangle |k\rangle \langle k^{\prime }|\langle I|e^{-iq^{\prime }\;\cdot \;\hat{R}}|F\rangle |{\tilde{k}}^{\prime }\rangle \delta \left(E_{F}-E_{I}-E\right),$$

the probability for a “click” in the detector can be written as [8,45,48,49,50]

(4) $$p=\;\int \int \int \int \;\sum_{x}e^{i(q-q^{\prime })\;\cdot \;x}\psi_{\mathrm{out}}{\left(\tilde{k}\right)}^{*}\psi_{\mathrm{out}}\left({\tilde{k}}^{\prime }\right)\frac{S(q,q^{\prime },E)}{q^{2}{q^{\prime }}^{2}}\psi_{\mathrm{in}}\left(k\right)\psi_{\mathrm{in}}{\left(k^{\prime }\right)}^{*}dkdk^{\prime }d\tilde{k}d{\tilde{k}}^{\prime },$$

where the $\sum_{x}e^{i(q-q^{\prime })\;\cdot \;x}$ stems from the summation over all atoms (of the investigated species) in the sample and the MDFF is taken to be the MDFF of a single such atom located at the origin.

Specific expressions for the MDFF for various models under different conditions and approximations are well known (see, e.g., [7,49,52]), but their details will be irrelevant for the majority of our derivation for which we will keep the general expression $S(q,q^{\prime },E)$.

Using the Bloch wave formalism [8,36,53,54,55], the three-beam incident wavefunction and the two-beam outgoing wave function can be written as

(5) $$\begin{array}{cc} |\psi_{\mathrm{in}}\rangle & =\sum_{j\in \{1,2,3\}}\sum_{g\in \{-G,0,G\}}C_{j,0}^{*}C_{j,0}|\chi +\gamma_{j}n+g\rangle \end{array}$$

(6) $$\begin{array}{cc} |\psi_{\mathrm{out}}\rangle & =\sum_{l\in \{1,2\}}\sum_{h\in \{0,G\}}{\tilde{C}}_{l,0}^{*}e^{-i{\tilde{\gamma }}_{l}t}{\tilde{C}}_{l,h}|\tilde{\chi }+\tilde{\gamma_{l}}\tilde{n}+h\rangle , \end{array}$$

where j,l are the Bloch wave indices, g,h run over the diffraction spots, the $C_{j,g}$ are the Bloch wave coefficients, the $\gamma_{j}$ are the so-called anpassung, n is the surface normal vector, *t* is the sample thickness, and $\chi ,\tilde{\chi }$ are the wave vectors of the incident and outgoing plane waves, respectively.

The derivation of the EMCD effect can be found in Appendix A. The final expression is

(7) $$\eta =\frac{A\sin^{2}\left(\kappa t\right)-B\sin^{2}\left(\kappa^{\prime }t\right)}{t+C\sin(2\kappa t)}\;\cdot \;\frac{ℑ[S\left(q_{1},q_{2},E\right)]}{S(q_{1},q_{1},E)},$$

where *t* is the sample thickness and the coefficients $A,B,C,\kappa ,\kappa^{\prime }$ are defined in Equation (A18) (with Equations (A1) and (A3)).

**Figure 1:**
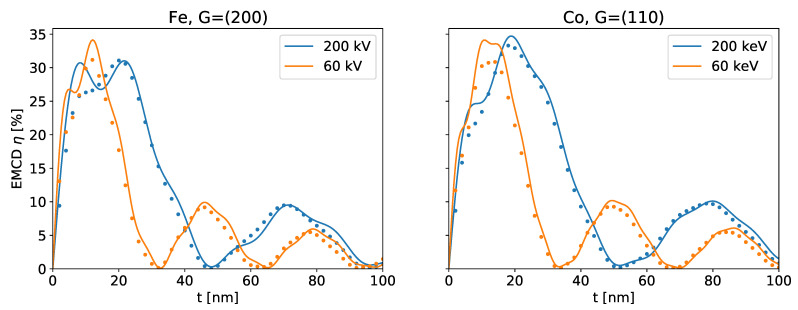
Comparison of the thickness dependence of the EMCD effect η predicted by Equation (7) (solid lines) and by the “bw” software using Equation (4) (dotted lines) for different acceleration voltages for bcc Fe and hcp Co.

Figure 1 shows a comparison of the thickness dependence predicted by Equation (7) and a full simulation based on Equation (4) for some typical, simple magnetic samples. Owing to the approximations made in the derivation, there naturally are some small differences (which are more pronounced at small thicknesses), but they are well within typical experimental uncertainties.

Two main conclusions about the thickness-variation of the EMCD effect can be drawn from Equation (7). On the one hand, the numerator nicely shows the oscillatory nature of the effect. On the other hand, the denominantor clearly implies that the strength of the EMCD effect decreases approximately as 1/t.

**Figure 2:**
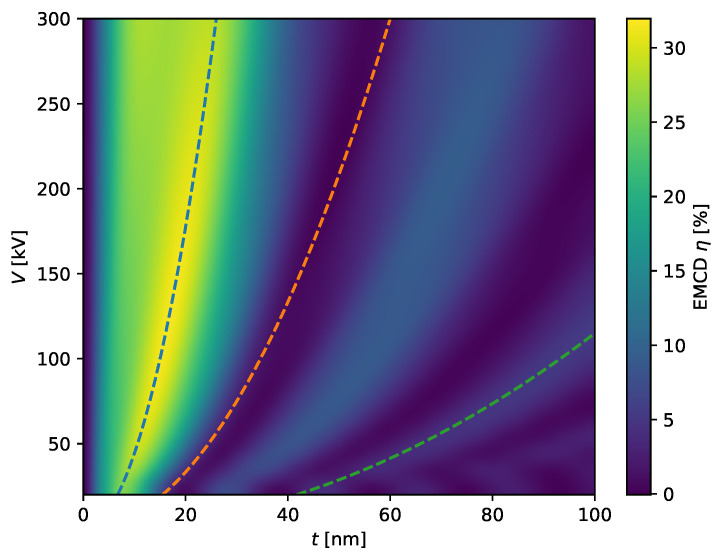
EMCD effect η for various acceleration voltages *V* and thicknesses *t* for bcc Fe as simulated with “bw”. The dashed lines show (arbitrary) curves with $t\propto \sqrt{V}$ as guides for the eye.

The numerator is composed of two oscillations with different amplitudes (A,B) and the frequencies

(8) $$\kappa =\frac{\gamma_{1}-\gamma_{2}}{2}=\frac{\sqrt{{\left(|G\right|}^{2}-U_{2G}{)}^{2}+8U_{G}^{2}}}{4\chi \;\cdot \;n}\;\mathrm{and}\;\kappa^{\prime }=\frac{{\tilde{\gamma }}_{1}-{\tilde{\gamma }}_{2}}{2}=\frac{U_{G}}{2\tilde{\chi }\;\cdot \;\tilde{n}}$$

which are closely related to the extinction distances for the incident and outgoing beams. As the wavevectors $\chi ,\tilde{\chi }$ scale with the square root of the acceleration voltage $\sqrt{V}$, the frequencies of the oscillations of the EMCD effect scale with $1/\sqrt{V}$. This is corroborated by Figure 2.

Both the oscillations and the 1/t decay can be understood from the fact that EMCD is essentially an interferometry experiment. As such, it crucially depends on the relative phases of the different density matrix components after traversing the sample from the scattering center to the exit plane. Some scattering centers are positioned in a way that the resulting components contribute positively to the EMCD effect, other scattering centers are positioned such that their contribution to the EMCD effect is negative. As a result, there are alternating “bands” of atoms contributing positively and negatively [11], where the size of the bands is related to the extinction length. With increasing thickness, more and more alternating bands appear—the non-magnetic signal increases linearly with *t*, but the magnetic EMCD signal of all but one band averages out, ultimately resulting in a 1/t behavior of the relative EMCD effect.

Our theoretical results have several important implications. First, the EMCD effect can indeed be recorded at a wide variety of acceleration voltages as already proposed on numerical grounds in [42], thereby enabling magnetization-dependent measurements. Second, the thickness dependence scales with 1/t, thus necessitating thin samples. Third, for a given sample thickness in the region of interest, a candidate for the optimal high tension yielding the maximal EMCD effect can easily be identified based on any existing simulation and the $\sqrt{V}$ scaling behavior (note, however, that other effects such as multiple plasmon scattering can put further constraints on the useful range of sample thicknesses, particularly at very low voltages).

### 2.2. Experiments

**Figure 3:**
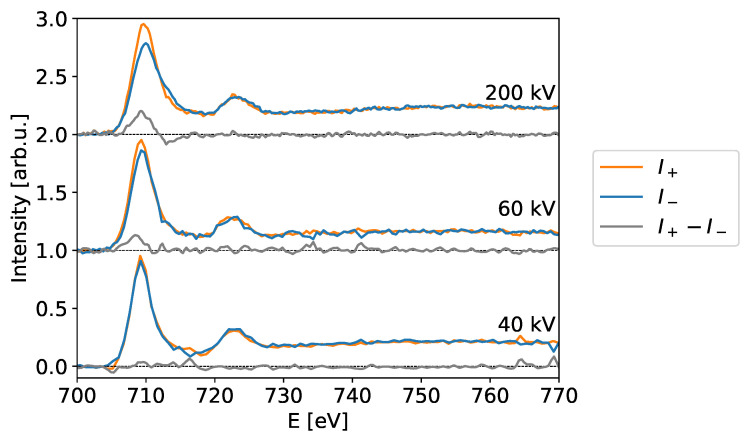
EMCD spectra for different acceleration voltages (as indicated) after background subtraction and post-edge normalization using the Fe L-edge in Magnetite tilted to a (4 0 0) systematic row condition. The sample-thickness was determined to be t≈35nm for the 40kV and 60kV measurement positions and t≈45nm for the 200kV measurement position.

**Figure 4:**
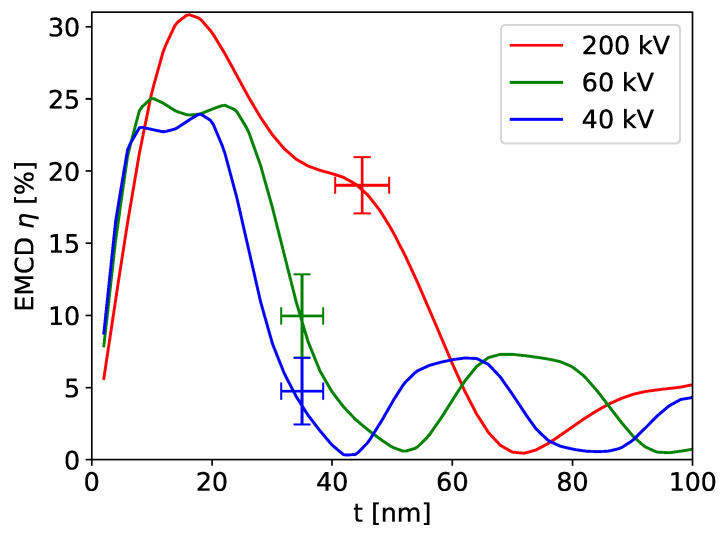
Comparison between numerical EMCD simulations (“bw”, solid curves) and experiments (points) for Magnetite for three different acceleration voltages. For the experimental points, η was calculated from the data in Figure 3 according to Equation (9), the measured thickness values are given in the caption of Figure 3, and the error bars were determined as described in [57,58].

To corroborate our theoretical finding, we performed experiments at various high tensions to compare to the simulations. The experiments were performed on a ferrimagnetic magnetite (Fe_3_O_4_) sample [56], which has the advantage over pure Fe that it is unaffected by oxidation (it may, however, be partially reduced to Wüstite by prolonged ion or electron irradiation). The individual recorded spectra are shown in Figure 3. It is clearly visible that the EMCD effect changes with the high tension as predicted in Section 2.1. A quantitative comparison between the calculations and the experiments is shown in Figure 4 and shows excellent agreement.

## 3. Discussion

Although Equation (7) is—to our knowledge—the first complete, analytical, closed form predicting the EMCD effect, several assumptions and approximations were made in its derivation. As such it is no replacement for full simulations with sophisticated software packages if ultimate accuracy is vital. Nevertheless, it can be a good starting point for EMCD investigations, and it helps elucidating the underlying physical principles and understanding the effects the experimental parameters have on EMCD. In this section, we will discuss the limits of the theoretical derivation based on the approximations made.

Assumption one deals with the scattering geometry and the crystal structure. The incident three-beam and outgoing two-beam case is the simplest approximation taking into account elastic scattering both before and after an inelastic scattering event. Adding more beams to the calculation can, of course, improve the results somewhat. However, the effect was found to be very small and well within typical experimental uncertainties [11], owing primarily to the $1/q^{2}{q^{\prime }}^{2}$ term in Equation (4) (any additional beams would give rise to much longer q vectors). The crystal structure was assumed to be centro-symmetric, resulting in $U_{G}=U_{-G}$. While this limits the applicability of the formula to relatively simple crystals, very complex, non-symmetric crystals will likely violate some of the other assumptions as well. In addition, the constraints implied by centro-symmetry are necessary in the first place to arrive at a reasonably simple final formula.

Assumption two requires the sample’s surface to be essentially perpendicular to the beam direction. This requirement is necessary to avoid complex phase factors down the line. A small tilt of up to a few degrees is not expected to cause any major issues, and larger tilts of ≳45 ${}^{\circ }$C are not recommended (and often not even possible) in practice anyway.

Assumption three requires the inelastic scattering process to be invariant under rotations around the optical axis by integer multiples of 90${}^{\circ }$. Strong anisotropy would lead to a distinct directional dependence of the MDFF [48,59,60], thereby making it impossible to reason about the intensities at the various detector positions. In such cases, however, the classical EMCD setup would fail to properly measure the magnetic properties anyway. In addition, assumption three states $q_{e}\ll \left|G\right|$, which implies $ℑ\left[S\left(q_{1},q_{2},E\right)\right]\ll ℜ\left[S\left(q_{1},q_{2},E\right)\right]$ in dipole approximation [11,61]. This is fulfilled reasonably well for typical EMCD experiments (for example, for Fe(2 0 0), $|G|\approx 7{\mathrm{nm}}^{-1}$; for the Fe L-edge, $q_{e}\approx 0.8{\mathrm{nm}}^{-1}$ at 200 keV and $q_{e}\approx 1.5{\mathrm{nm}}^{-1}$ at 40 keV).

Assumption four requires the investigated atoms to be distributed homogeneously and fulfill the condition G·x=2mπ. The homogeneity requirement excludes involved situations such as multi-layer systems and ultimately allows to replace the sum over all atoms by an integral over the sample thickness. In practice, homogeneity is facilitated by tilting into a systematic row condition and probing a large area of the sample, as a large probed volume and a (small) tilt mean that some atoms can be found in each of the investigated lattice planes at any depth *z*.

The condition G·x=2mπ∀x is perhaps the most severe limitation as it implies that all atoms fall exactly onto one of the probed set of lattice planes. This excludes, e.g., G=(100) for Fe (which is forbidden anyway), or G=(100) for Co, as for these, only some (for Fe) or none (for Co) of the atoms fulfill the condition. The reason for requiring G·x=2mπ is that it implies that phase factors of the form exp(iG·x) are all 1. If that is not the case, different phases have to be applied to different components, thereby reducing the EMCD effect [41]. Hence, choosing a G vector not fulfilling the condition is unfavorable anyway.

As can be seen from Figure 1, Equation (7) reproduces sophisticated numerical simulations quite well for reasonably simple samples despite all approximations. The strongest deviations can be found for small *t*, as can be expected. For larger sample thicknesses and, consequently, many atoms, small differences that might arise for individual atoms tend to average out.

## 4. Materials and Methods

The numerical simulations were performed using the “bw” code [11], a software package for calculating EELS data based on Bloch waves and the MDFF. The crystal structure data for magnetite was taken from [62], all other crystallographic data was taken from the EMS program (version 4.5430U2017) [63].

The wedge-shaped magnetite sample was prepared by a FEI Quanta 200 3D DBFIB (FEI Company, Hillsboro, OR, USA) from a high-quality, natural single crystal purchased from SurfaceNet GmbH (Rheine, Germany) [64] and subsequently thinned and cleaned using a Technoorg Linda Gentlemill.

**Figure 5:**
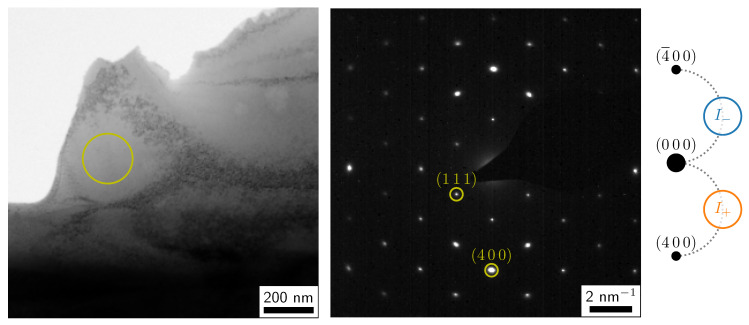
TEM bright-field overview image (**left**), corresponding diffraction pattern in (0 1 $\bar{1}$) zone axis (**middle**) and schematic of the EMCD measurement positions in systematic row condition (**right**). The sample position used for the EMCD experiments is marked by a yellow circle in the bright-field image, the positions for $I_{+}$ and $I_{-}$ are marked by the orange and blue circles. Both the image and the diffraction pattern were recorded at 200kV. Note that the weak, kinematically forbidden (2 0 0) reflections can be attributed to double diffraction [36] in the thicker part of the sample visible at the bottom of the bright-field image; they are negligible in the thin part of the sample used for the EMCD measurements.

The EMCD measurements were performed on a FEI Tecnai T20 (FEI Company, Hillsboro, OR, USA) equipped with a LaB_6_ gun and a Gatan GIF 2001 spectrometer (Gatan Inc., Pleasanton, CA, USA). The system has an energy resolution (full width at half maximum) of 1.1 eV at 200 kV which improves down to 0.3 eV at 20 kV [65]. First, a suitable sample position with a sample thickness around 40 nm and an easily recognizable, distinctly-shaped feature nearby was found and the sample was oriented in systematic row condition including the (4 0 0) diffraction spot (see Figure 5). At each high tension, the instrument was carefully aligned, the sample position was readjusted, the EMCD experiment was performed, and a thickness measurement was taken. Both the convergence and the collection semi-angle were approximately 3 mrad [58].

For data analysis, all spectra were background-subtracted using a pre-edge power-law fit and normalized in the post-edge region. The EMCD effect was calculated based on the L_3_-edge maxima according to the formula [9,58]

(9) $$\eta =\frac{I_{+}-I_{-}}{\frac{I_{+}+I_{-}}{2}}.$$

The errors were estimated as described in [57,58].

## 5. Conclusions

In this work, we have derived an analytical formula for predicting the EMCD effect, taking into account elastic scattering both before and after inelastic scattering events. This formula not only helps elucidate the physics underlying EMCD, it also allows to directly predict the influence of various parameters on the EMCD effect. In particular, we have focused on the acceleration voltage *V* and on the thickness *t*. We showed that the periodicity of the EMCD effect scales with $\sqrt{V}$, while its total intensity decreases as 1/t. In addition, we have performed experiments at different acceleration voltages to corroborate these predictions. Our results will not only help to optimize the EMCD effect for a given sample thickness by tuning the high tension accordingly, it will also pave the way for magnetization-dependent measurements by employing different magnetic fields in the objective lens at different acceleration voltages.

## Data Availability

Data is contained within the article.

## Abbreviations

The following abbreviations are used in this manuscript:

DDSCS	Double-differential scattering cross-Section
EMCD	Energy-loss magnetic chiral dichroism
EELS	Electron energy-loss spectrometry
MDFF	Mixed dynamic form factor
TEM	Transmission electron microscopy
XAS	X-ray absorption spectroscopy
XMCD	X-ray magnetic circular dichroism

## Appendix A. Derivation of the EMCD Effect

In the following, we will extensively use the abbreviations

(A1) $$\begin{array}{cc} \alpha & =\frac{U_{G}}{2\chi \;\cdot \;n}\;\tilde{\alpha }=\frac{U_{G}}{2\tilde{\chi }\;\cdot \;\tilde{n}} \end{array}$$

(A2) $$\begin{array}{cc} V & =\frac{U_{2G}-{\left|G\right|}^{2}}{2U_{G}} \end{array}$$

(A3) $$\begin{array}{cc} W & =\frac{\sqrt{{\left(|G\right|}^{2}-U_{2G}{)}^{2}+8U_{G}^{2}}}{2U_{G}}=\sqrt{V^{2}+2}, \end{array}$$

where the $U_{g}$ are the Fourier coefficients of the crystal potential $V\left(r\right)=\frac{h^{2}}{2me}\sum_{g}U_{g}e^{2\pi ig\;\cdot \;r}$ with Planck’s constant *h*, electron mass *m* and elementary charge *e*. We note in passing that in the present case, $U_{G}=U_{G}^{*}=U_{-G}$.

With these abbreviations and the assumptions mentioned above, the Bloch wave parameters can be calculated analytically and take the form

(A4) $$\begin{array}{cccccc} \gamma_{1} & =\alpha (V+W) & \gamma_{2} & =\alpha (V-W) & \gamma_{3} & =-\alpha \;\cdot \;\frac{{\left|G\right|}^{2}+U_{2G}}{U_{G}}\\ C_{1,-G} & =\frac{1}{\sqrt{{\left|V-W\right|}^{2}+2}} & C_{2,-G} & =\frac{1}{\sqrt{{\left|V+W\right|}^{2}+2}} & C_{3,-G} & =-\frac{1}{\sqrt{2}}\\ C_{1,0} & =-\frac{V-W}{\sqrt{{\left|V-W\right|}^{2}+2}} & C_{2,0} & =-\frac{V+W}{\sqrt{{\left|V+W\right|}^{2}+2}} & C_{3,0} & =0\\ C_{1,G} & =\frac{1}{\sqrt{{\left|V-W\right|}^{2}+2}} & C_{2,G} & =\frac{1}{\sqrt{{\left|V+W\right|}^{2}+2}} & C_{3,G} & =\frac{1}{\sqrt{2}} \end{array}$$

for $|\psi_{\mathrm{in}}\rangle$ and

(A5) $$\begin{array}{cccc} {\tilde{\gamma }}_{1} & =\tilde{\alpha } & {\tilde{\gamma }}_{2} & =-\tilde{\alpha }\\ {\tilde{C}}_{1,0} & =\frac{1}{\sqrt{2}} & {\tilde{C}}_{2,0} & =\frac{1}{\sqrt{2}}\\ {\tilde{C}}_{1,G} & =\frac{1}{\sqrt{2}} & {\tilde{C}}_{2,G} & =-\frac{1}{\sqrt{2}} \end{array}$$

for $|\psi_{\mathrm{out}}\rangle$.

Inserting Equations (5) and (6) into Equation (4), evaluating the integrals, collecting all terms with the same Bloch wave index, and neglecting the weak dependence of $S\left(q,q^{\prime },E\right)/\left(q^{2}{q^{\prime }}^{2}\right)$ on $j,j^{\prime },l,l^{\prime }$ [8,41,55] yields

(A6) $$p=\sum_{x}\sum_{g,g^{\prime },h,h^{\prime }}D_{g}D_{g^{\prime }}^{*}{\tilde{D}}_{h}^{*}{\tilde{D}}_{h^{\prime }}e^{i(g-g^{\prime }-h+h^{\prime })\;\cdot \;x}\frac{S(q,q^{\prime },E)}{q^{2}{q^{\prime }}^{2}}$$

with

(A7) $$D_{g}=\sum_{j}C_{j,0}^{*}C_{j,g}e^{i\gamma_{j}n\;\cdot \;x}\;{\tilde{D}}_{g}=\sum_{l}{\tilde{C}}_{l,0}^{*}e^{-i{\tilde{\gamma }}_{l}t}{\tilde{C}}_{l,h}e^{i{\tilde{\gamma }}_{j}\tilde{n}\;\cdot \;x}$$

and

(A8) $$q=\Delta \chi +g-h\;q^{\prime }=\Delta \chi +g^{\prime }-h^{\prime }\;\Delta \chi =\chi -\tilde{\chi }.$$

Direct summation results in

(A9) $$\begin{array}{ccc} D_{-G} & = & D_{G}=\frac{i}{W}e^{i\alpha Vn\;\cdot \;x}\sin\left(\alpha Wn\;\cdot \;x\right)\\ & & D_{0}=e^{i\alpha Vn\;\cdot \;x}\left[\cos\left(\alpha Wn\;\cdot \;x\right)-\frac{iV}{W}\sin\left(\alpha Wn\;\cdot \;x\right)\right]\\ & & {\tilde{D}}_{0}=\cos(\tilde{\alpha }\left(\tilde{n}\;\cdot \;x-t\right))\\ & & {\tilde{D}}_{G}=i\sin(\tilde{\alpha }\left(\tilde{n}\;\cdot \;x-t\right)). \end{array}$$

Performing the complete sums over $g,g^{\prime },h,h^{\prime }$ in Equation (A6) produces very many terms, some of which are very small. This can be understood from the fact that Δχ·G=±G/2 in the chosen setup. Therefore, Δχ and Δχ−G have the same magnitude, whereas Δχ+G and Δχ−2G are significantly larger. Owing to the $1/q^{2}{q^{\prime }}^{2}$ term, large q are strongly suppressed. Hence, only the combinations g−h=0 and g−h=−G are retained (the same applies to the primed versions as well). Hence, we end up with two distinct q vectors, namely

(A10) $$q_{1}=\Delta \chi \;\mathrm{and}\;q_{2}=\Delta \chi -G.$$

Note that, due to the symmetry of the setup $q_{1}=|q_{1}\left|=\right|q_{2}|=q_{2}$.

Using $S\left(q,q^{\prime },E\right)=S{\left(q^{\prime },q,E\right)}^{*}$ [45], Equation (A6) now takes the form

(A11) $$\begin{array}{cc} p=\frac{1}{q_{1}^{4}}\sum_{x} & [{\left|D_{0}{\tilde{D}}_{0}^{*}+D_{G}{\tilde{D}}_{G}^{*}\right|}^{2}S\left(q_{1},q_{1},E\right)+\\ \; & {\left|D_{-G}{\tilde{D}}_{0}^{*}+D_{0}{\tilde{D}}_{G}^{*}\right|}^{2}S\left(q_{2},q_{2},E\right)+\\ \; & \left.2ℜ\left[\left(D_{0}{\tilde{D}}_{0}^{*}+D_{G}{\tilde{D}}_{G}^{*}\right)\left(D_{-G}^{*}{\tilde{D}}_{0}+D_{0}^{*}{\tilde{D}}_{G}\right)e^{iG\;\cdot \;x}S\left(q_{1},q_{2},E\right)\right]\right]\\ =\frac{1}{q_{1}^{4}} & \left.A_{11}S\left(q_{1},q_{1},E\right)+A_{22}S\left(q_{2},q_{2},E\right)+2ℜ\left[A_{12}S\left(q_{1},q_{2},E\right)\right]\right]\\ =\frac{1}{q_{1}^{4}} & \left.\left(A_{11}+A_{22}\right)S\left(q_{1},q_{1},E\right)+2ℜ\left[A_{12}S\left(q_{1},q_{2},E\right)\right]\right]. \end{array}$$

In the last line, the four-fold rotational symmetry was used, i.e., $S\left(q_{1},q_{1},E\right)=S\left(q_{2},q_{2},E\right)$ since $q_{2}={\hat{C}}_{4}\left[q_{1}\right]$ with ${\hat{C}}_{4}$ as the operator performing a 90${}^{\circ }$ rotation around the optical axis.

To calculate the probability for a “click” in the detector at the second EMCD position, we have to replace $q_{1}\mapsto {\hat{C}}_{4}^{3}\left[q_{1}\right]={\hat{C}}_{4}^{2}\left[q_{2}\right]$ and $q_{2}\mapsto {\hat{C}}_{4}\left[q_{2}\right]={\hat{C}}_{4}^{2}\left[q_{1}\right]$. Owing to the assumed rotational symmetry of the MDFF, this replacement results in $S\left({\hat{C}}_{4}^{2}\left[q_{2}\right],{\hat{C}}_{4}^{2}\left[q_{1}\right],E\right)=S\left(q_{2},q_{1},E\right)=S{\left(q_{1},q_{2},E\right)}^{*}$ and hence

(A12) $$p^{\prime }=\frac{1}{q_{1}^{4}}\left[\left(A_{11}+A_{22}\right)S\left(q_{1},q_{1},E\right)+2ℜ\left[A_{12}S{\left(q_{1},q_{2},E\right)}^{*}\right]\right].$$

Thus, the quotient EMCD effect is

(A13) $$\eta =2\;\cdot \;\frac{p-p^{\prime }}{p+p^{\prime }}=2\;\cdot \;\frac{-2ℑ\left[A_{12}\right]ℑ\left[S\left(q_{1},q_{2},E\right)\right]}{\left(A_{11}+A_{22}\right)S\left(q_{1},q_{1},E\right)+2ℜ\left[A_{12}\right]ℜ\left[S\left(q_{1},q_{2},E\right)\right]}$$

Assuming that the scattering vectors were chosen such that $S(q_{1},q_{2},E)$ is purely imaginary (technically, (in dipole approximation) this occurs slightly inside the Thales circle where $q_{y}^{2}=G^{2}/4-q_{e}^{2}$; as $q_{e}\ll G$ in typical EMCD experiments, the real part of $S(q_{1},q_{2},E)$, which is of the order $q_{e}^{2}$, can be neglected compared to $S(q_{1},q_{1},E)$, which is of the order of $G^{2}/2$), this can be simplified further to

(A14) $$=-4\;\cdot \;\frac{ℑ\left[A_{12}\right]}{A_{11}+A_{22}}\;\cdot \;\frac{ℑ[S\left(q_{1},q_{2},E\right)]}{S(q_{1},q_{1},E)}.$$

The coefficients can be calculated directly as

(A15) A11+A22=∑x1−1W2sin2(αWn·x)ℑ[A12]=∑x121−3W2sin2(αWn·x)sin(2α˜(n˜·x−t))=∑x−1Wsin(2αWn·x)cos(2α˜(n˜·x−t)).

with the assumptions 2 and 4, the dot products can be evaluated and the sums can be replaced by integrals over *z*, yielding

(A16) $$\begin{array}{cc} A_{11}+A_{22} & =t\left(1-\frac{1}{2W^{2}}\right)+\frac{\sin(2tW\alpha )}{4W^{3}\alpha }\\ ℑ[A_{12}] & =\frac{1}{4(W^{2}\alpha^{2}-{\tilde{\alpha }}^{2})}\left[-\left(2\alpha +\frac{3\tilde{\alpha }}{W^{2}}\right)\sin^{2}\left(\alpha Wt\right)\right.\\ \; & \left.+\left(\frac{\left(3-2W^{2}\right)\alpha^{2}}{\tilde{\alpha }}+2\left(\alpha +\tilde{\alpha }\right)\right)\sin^{2}\left(\tilde{\alpha }t\right)\right] \end{array}$$

Hence the full formula for the EMCD effect reads

(A17) $$\begin{array}{cc} \eta \; & =\frac{4W^{3}\alpha }{\left(W^{2}\alpha^{2}-{\tilde{\alpha }}^{2}\right)}\frac{\left[\left(2\alpha +\frac{3\tilde{\alpha }}{W^{2}}\right)\sin^{2}\left(\alpha Wt\right)-\left(\frac{\left(3-2W^{2}\right)\alpha^{2}}{\tilde{\alpha }}+2\left(\alpha +\tilde{\alpha }\right)\right)\sin^{2}\left(\tilde{\alpha }t\right)\right]}{2W\left(2W^{2}-1\right)\alpha t+\sin\left(2tW\alpha \right)}\;\cdot \;\frac{ℑ[S\left(q_{1},q_{2},E\right)]}{S(q_{1},q_{1},E)}\\ \; & =\frac{A\sin^{2}\left(\kappa t\right)-B\sin^{2}\left(\kappa^{\prime }t\right)}{t+C\sin(2\kappa t)}\;\cdot \;\frac{ℑ[S\left(q_{1},q_{2},E\right)]}{S(q_{1},q_{1},E)} \end{array}$$

with

(A18) $$\begin{array}{cc} A & =C\;\cdot \;\frac{4\kappa \kappa^{\prime }}{\kappa^{2}-{\kappa^{\prime }}^{2}}\left(2W\frac{\kappa }{\kappa^{\prime }}+3\right)\\ B & =C\;\cdot \;\frac{4\kappa \kappa^{\prime }}{\kappa^{2}-{\kappa^{\prime }}^{2}}\left(2W\frac{\kappa }{\kappa^{\prime }}+\frac{3\kappa^{2}}{{\kappa^{\prime }}^{2}}+2W^{2}\left(1-\frac{\kappa^{2}}{{\kappa^{\prime }}^{2}}\right)\right)\\ C & =\frac{1}{2\kappa (2W^{2}-1)}\\ \kappa & =\alpha W=\frac{\gamma_{1}-\gamma_{2}}{2}\\ \kappa^{\prime } & =\tilde{\alpha }=\frac{{\tilde{\gamma }}_{1}-{\tilde{\gamma }}_{2}}{2}. \end{array}$$
